# Random genome reduction coupled with polyhydroxybutyrate biosynthesis to facilitate its accumulation in *Escherichia coli*

**DOI:** 10.3389/fbioe.2022.978211

**Published:** 2022-08-29

**Authors:** Shuai Ma, Tianyuan Su, Jinming Liu, Qian Wang, Quanfeng Liang, Xuemei Lu, Qingsheng Qi

**Affiliations:** State Key Laboratory of Microbial Technology, Shandong University, Qingdao, China

**Keywords:** random genome reduction, polyhydroxybutyrate, glucose utilization, growth, *E. coli*

## Abstract

Genome reduction has been emerged as a powerful tool to construct ideal chassis for synthetic biology. Random genome reduction couple genomic deletion with growth and has the potential to construct optimum genome for a given environment. Recently, we developed a transposon-mediated random deletion (TMRD) method that allows the random and continuous reduction of *Escherichia coli* genome. Here, to prove its ability in constructing optimal cell factories, we coupled polyhydroxybutyrate (PHB) accumulation with random genome reduction and proceeded to reduce the *E. coli* genome. Five mutants showed high biomass and PHB yields were selected from 18 candidates after ten rounds of genome reduction. And eight or nine genomic fragments (totally 230.1–270.0 Kb) were deleted in their genomes, encompassing 4.95%–5.82% of the parental MG1655 genome. Most mutants displayed better growth, glucose utilization, protein expression, and significant increase of electroporation efficiency compared with MG1655. The PHB content and concentration enhanced up to 13.3%–37.2% and 60.2%–102.9% when batch fermentation was performed in M9-glucose medium using the five mutants. Particularly, in mutant H16, lacking 5.28% of its genome, the increase of biomass and PHB concentration were more than 50% and 100% compared with MG1655, respectively. This work expands the strategy for creating streamlined chassis to improve the production of high value-added products.

## Introduction

As for engineered microbes cultured in stable laboratory conditions, many genomic regions are considered redundant, e.g., stress response genes, insertion sequence elements, transposons, and so forth, and may even increase mutagenesis and cause the burden on cellular growth and metabolism (Kurokawa and Ying, 2020). Synthetic biologists have been devoting to designing and modifying microorganisms to function as cell factories in which harbored a streamlined genome for full functionality and a metabolic network able to more effectively synthesize the desired products (Chi et al., 2019).

Genome reduction has been developed as a feasible strategy to obtain streamlined chassis for enhanced production of high value-added products. In many bacteria, moderate genome reduction by removing multiple dispensable genomic regions has been proved to be able to optimize metabolic pathways, enhance the heterologous protein productivity, and improve the growth and physiological performance (Kolisnychenko et al., 2002; Posfai et al., 2006; Mizoguchi et al., 2008; Komatsu et al., 2010; Wang et al., 2018; Liang et al., 2020; Qiao et al., 2020; Zhang et al., 2020). For instance, the MDS (multiple-deletion series) strains, lacking 8.11%–14.3% of genome sequences (including mobile elements, virulence genes, and so forth) in *Escherichia coli* MG1655, showed some favorable properties, normal growth, increased electroporation efficiency and protein expression level (Kolisnychenko et al., 2002; Posfai et al., 2006). *E. coli* MGF-01 lost 22% of the original genomic sequence and achieved a 2.4-fold increase in L-threonine production (Mizoguchi et al., 2008). After removal of 16.8%–18.5% of the original genome sequences (the large left subtelomeric region), a series of genome-minimized *Streptomyces avermitilis* strains were obtained in which higher heterologous expression of three secondary metabolites, streptomycin, cephamycin C and pladienolide were achieved while leaving growth and primary metabolism unaffected (Komatsu et al., 2010). Deletion of the genomic islands, phage-related genes, and some other nonessential genomic regions (accounting for 1.2%–4.12% of the total genome sizes) facilitated the transformation efficiency, heterologous protein expression, and polyhydroxyalkanoate production in *Pseudomonas putida* (Wang et al., 2018; Liang et al., 2020). The increase of multi-stress tolerance and nisin immunity was achieved in *Lactococcus lactis* when deleting 19.7-kb of prophage-related fragments (Qiao et al., 2020). The growth and surfactin production of *Bacillus amyloliquefaciens* was increased by deleting ∼4.18% of genome sequences (Zhang et al., 2020). The DT (deletions of transposons) series genome-reduced *Schlegelella brevitalea* mutants were rationally constructed by sequential deletions 0.3%–4.9% of its genome, including forty-four transposases, two prophage-like regions, and seven genomic islands, and showed better growth characteristics with alleviated cell autolysis, and improved yields of six proteobacterial natural products (Liu et al., 2021).

Common methods to construct genome-simplified chassis were based on rational design and analysis of the essential DNA elements, followed by the targeted deletion of large dispensable DNA fragments *via* seamless genome deletion method (Choe et al., 2016). The limitation of rational design strategy is the difficulty to judge the necessity of targeted regions and systematic investigate the interrelation of genome deletion and phenotype due to incomplete knowledge of complex genome function and interactions. That result in decreased growth, perturbations of the physiological properties, and unstable productivity of recombinant protein or desired products in genome-reduced strains obtained by rational design (Iwadate et al., 2011; Li et al., 2016; Tsuchiya et al., 2018). To overcome this limitation, researchers have proposed random genome reduction strategies (Suzuki et al., 2008; Vernyik et al., 2020; Shaw et al., 2021)that can couple genomic deletion with cellular growth, generate favorable deletion combinations in genome, and screen cells with fitness in different environment. To date, some random genome reduction methods have been completed to the proof-of-concept stage, but they are not efficient enough to construct desired chassis cells for application (Suzuki et al., 2008; Vernyik et al., 2020; Shaw et al., 2021). In previous study, we developed a random and continuous genome reduction method, named as “transposon-mediated random deletion (TMRD),” which enables the reduction of *E. coli* genome by random deletion accompanied by simultaneous enrichment of the cells with environment fitness (Ma et al., 2022). We have proved the feasibility of this method and completed five rounds of genome reduction using *E. coli* MG1655 in rich LB medium (Ma et al., 2022), but it remains unclear that whether other appropriate conditions can be employed to create optimal cell factories for different application purposes.

Polyhydroxybutyrate (PHB) are a class of biopolymers that can be accumulated in microorganism cells under unfavorable growth conditions as both an energy and carbon store (Li et al., 2020). The PHB competent cells can be easily screened by Nile red staining as the legible red colonies are produced when PHB are combined with Nile red dye (Li et al., 2020). Here, we combined the PHB biosynthesis with random deletion and aimed at investigating whether improved PHB accumulation by engineered *E. coli* can be accomplished by random genome reduction. To this end, we continued to reduce the *E. coli* MG1655 genome *via* TMRD method while screening the PHB competent cells by Nile red assay. The mutants with high PHB accumulation were chosen to determine the genomic deletions, growth features, glucose utilization, transformation efficiency, protein expression level, and PHB accumulation compared with the original strain.

## Materials and methods

### Bacterial strains, culture medium, and reagents

All strains used in this study were shown in Table 1. The *E. coli* K-12 strain DH5α was used as the host strain for molecular cloning and manipulation of plasmids. The parental *E. coli* MG1655 was used as the control strain in all experiments. Other strains used for genome reduction were same as previous report (Ma et al., 2022).

**TABLE 1:** Strains and plasmids used in this work.

Strains	Genotypes	Source
*E. coli* DH5α	Strain K-12, F^−^ *end*A1 thi-1 *gln*V4 4*rel*A1 *rec*A1 *deo*R *gyr*A96 *nup*G *φ80dlac*ZΔM15 Δ(*lac*ZYA*-arg*F) U169 *hsd*R17 (*rk* ^*-*^, *mk* ^*+*^) *λ* ^*-*^	Invitrogen
*E. coli* MG1655	F- lambda- *ilv*G- *rfb*-50 *rph*-1	Invitrogen
H2	Mutants. Random deletion of ∼270 Kb from MG1655	In this study
H5	Mutants. Random deletion of ∼230 Kb from MG1655	In this study
H16	Mutants. Random deletion of ∼245 Kb from MG1655	In this study
H19	Mutants. Random deletion of ∼261 Kb from MG1655	In this study
H20	Mutants. Random deletion of ∼236 Kb from MG1655	In this study
Plasmids	Descriptions	Source
pUC19	Cloning vector, pUC origin, Ap^*R*^	Norrander et al. (1983)
pACYAC184	Cloning vector, P15A origin, Cm^*R*^	Rose (1988)
pKTRED	pSC101 origin, containing Redαβγ recombinase system, Spc^*R*^	Ma et al. (2022)
pBHR68	pUC origin, *phb*CAB operon from *Ralstonia eutropha,* Ap^R^	Dila et al. (1990)
pBAC11rhi	Sing-copy bacterial artificial chromosome with a 92-kb fragment insertion, Cm^*R*^	Ma et al. (2022)
pACYC-GFP	pACYAC184 containing GFP gene	In this study
p15A-PHB	P15A origin, *phb*CAB genes from *R. eutropha,* Ap^R^	In this study
pSIP*pbh*CAB	pBHR68 containing stress-induced response fragment upstream of *rpo*S gene	In this study

If it was not mentioned specifically, all experiments involving in bacterial culture growth were conducted using standard LB rich medium (10 g/L tryptone, 5 g/L yeast extract, and 5 g/L NaCl). The M9 basal medium composed of (g/L): Na_2_HPO_4_.12H_2_O, 17.1; KH_2_PO4, 3.0; NaCl, 0.5; and NH_4_Cl, 1.0, supplemented with 1 mM MgSO_4_, 0.1 mM CaCl_2_, and 4 mg/L vitamin B1. M9-glucose media: M9 basal medium supplemented with appropriate glucose. When appropriate, ampicillin (Ap, 100 mg/L), chloramphenicol (Cm, 25 mg/L), isopropyl β-D-1-thiogalactopyranoside (IPTG, 1 mM) were supplemented into the cultures. Agar plates were prepared by adding 1.5% (w/v) agar to the media.

The Phanta Max Super-Fidelity DNA Polymerase (Vazyme, Qingdao, China) was utilized for the standard PCR amplifications. Green *Taq* Mix (Vazyme, Qingdao, China) was employed for colony PCR confirmation. MultiF Seamless Assembly Mix (RM20523) (Abclonal, Wuhan, China) was used for DNA fragment assembly and plasmid construction. Restriction enzymes and T4 DNA ligase were purchased from Thermo Fisher Biotech (Shanghai, China).

### Plasmids construction

All the plasmids and primers used in this study were list in Table 1 and Supplementary Table S1, respectively. The plasmids used for random genome reduction were same as previous study (Ma et al., 2022). p15A-PHB, a medium-copy-number plasmid expressing the *phb*CAB operon (from *Ralstonia eutropha*) (Choi and Lee, 1999) under the control of Lac promoter, were constructed by assembling two DNA fragments with 20 bp homologous bases *in vitro via* the Gibson assembly. The P15A module was amplified from pACYC184 using primers p15A-F and p15A-R. The Amp-phbCAB module was amplified from the pBHR68 (Choi and Lee, 1999) plasmid using primers PHB-F and Ap-R. The stress-induced region fragment was amplificated using a pair of primers SIP-F/SIP-R with *E. coli* genome as template. The PCR product was digested with *Xba* I and *Bam*H I and subcloned into vector pBHR68 (Choi and Lee, 1999) in replacement of Lac promoter segment and resulted in plasmid pSIP*phb*CAB. To construct the pACYC-GFP, the module J23104-gfp was synthesized by Tsingke Biotech (Beijing, China) and then assembled into the linearized plasmid pACYC184 cut by *Xba* I and *Sac* I.

### Random genome reduction for polyhydroxybutyrate accumulation

The genome reduction for PHB accumulation began with the genome-reduced library in the fifth cycle (R5) and followed the method of Ma et al. (2022) with minor modification. In detail, the cells from R5 library was used for the preparation of electrocompetent cells. Approximately 100 ng of plasmid (p15A-PHB) was electroporated into 50 μl of electrocompetent cells and spread onto LB agar plates with appropriate antibiotics. After incubation at 37°C overnight, all Ap^R^ colonies were collected and used as the starting library of genome reduction. Next, the random genome reduction was performed *via* TMRD method. To select the PHB competent cells, in each cycle, after inducing the generation of genomic deletions, the cells were spreading onto the M9-glucose agar plates supplemented with appropriate Nile red dye (Sigma, St. Louis, Mo United States) to give a final concentration of 2 mM; and then the plates were incubated at 37°C for 48 h to allow the color reaction to develop. Based on the color difference, the redder colonies that incurred genomic deletions were picked with the naked eye and used as the starting library for the next new cycle.

### Deletion efficiency, positions, and lengths analysis

Deletion efficiency was defined as the percentage of colonies loss of transposons against the total number of colonies analyzed and was measured following the previous method (Ma et al., 2022). The accumulative deletions in the genomes of the five selective mutants were identified by whole-genome sequencing and further verified *via* polymerase chain reaction (PCR) using genomic DNA as the template. The precise positions and lengths of the deletions in genome, and the base composition at junctions after repair of genome double-strands breaks, were confirmed by *Sanger* sequencing (Tsingke, Beijing, China). All PCR amplifications were performed using genomic DNA of the original *E. coli* MG1655 as the control.

Whole-genome sequencing and analysis were performed by GENEWIZ Biotech (Suzhou, China) using the Illumina NovaSeq platform. Using Cutadapt (v1.9.1) (Lindgreen, 2012) removed the sequcences of adaptors, PCR primers, content of N bases more than 10%, and bases of quality lower than 20. BWA (v0.7.17) (Li and Durbin, 2009) was employed to map clean data to the reference genome (NC_000913.3). Mapping results were processed by Picard (v2.25.7) for removal of duplication. The SNV and InDel was detected by GATK (V3.8.1) (McKenna et al., 2010) software. Annotations of single nucleotide variants (SNVs) and small insertions and deletions (InDels) were performed by Annovar (v21) (Odumpatta and Mohanapriya, 2020). The genomic structure variation was analyzed using Breakdancer (v1.1) (Chen et al., 2009) and CNVnator (v0.2.7) (Abyzov et al., 2011).

### Physiological traits assessment

To determine the maximal growth rate of the five mutants, cells were cultivated on a 24-well microplate with continuous shaking on a microplate reader (Epoch 2, BioTek, Winooski, VT, United States) for 16–24 h in LB rich medium and M9 minimal medium supplemented with 10 g/L glucose. The maximal growth rate (h^−1^) were measured and calculated as previously described (Ma et al., 2022). For the measurement of glucose utilization rate (g/L/h), overnight cultures were sub-cultured (1%, v/v) into 50 ml fresh M9 minimal medium supplemented with 10 g/L glucose, grown at 37°C, 220 rpm for 24 h. The glucose consumption (g/L) was quantified using a biosensor (SBA-40D, Biology Institute of Shandong Academy of Science, Shandong, China) (Feng et al., 2002) at the intervals of 1 or 2 h.

The preparation of electrocompetent cells and the assay of transformation efficiency were performed in accordance with the reported method (Ma et al., 2022). The transformation efficiency was defined as the number of colonies that formed on the corresponding antibiotic agar plates per microgram DNA (colony-forming units [cfu]/μg DNA). To assess the protein expression level, the genome-reduced strains carrying pACYC-GFP plasmids were cultivated on a 24-well microplate and grown at 37°C with continuous shaking for 24–48 h. The optical density (OD) at 600 nm and fluorescence intensity (FI, excitation at 485 nm and emission at 528 nm) were determined every 20 min using a Multi-Detection Microplate Reader (Synergy HT, Biotek, United States).

### Polyhydroxybutyrate fermentation analysis

For PHB production, 10 ml starter cultures were grown overnight in LB medium at 37°C with shaking at 220 rpm. Starter cultures (2%, v/v) were inoculated into shaken-flasks containing 50 ml M9 minimal medium supplemented with 30 g/L glucose and 2.0 g/L yeast extract, grown at 37°C and 220 rpm for 48 h. About 1 ml of fermentation broth were collected every 6 h. The properly diluted broth was directly used to measure OD_600_ by a spectrophotometer (UNICO 2000, United States) and glucose consumption (g/L) by a biosensor. At 48 h, all the cultures were collected and centrifuged at 8,000 rpm for 15 min. Then, the cell pellets were harvested and lyophilized for 12 h to analyze the final biomass. For extraction of PHB, 1 ml chloroform, 850 μl methanol, and 150 μl sulfuric acid (98%, w/w) were added to 15–20 mg weighed cells, and then the mixture was incubated at 100°C for 1 h. Next, 1 ml H_2_O was added into the mixture. After phage separation, the chloroform phase was used for gas chromatography analysis to quantify the intracellular PHB concentration (g/L) according to the method reported previously (Li et al., 2020).The biomass (g/L) was defined as the amount (dry weight) of cells per liter of culture broth. The PHB content (wt%) was defined as the percent ratio of PHB concentration to biomass.

## Results

### Coupled random genome reduction with polyhydroxybutyrate accumulation

**FIGURE 1:**
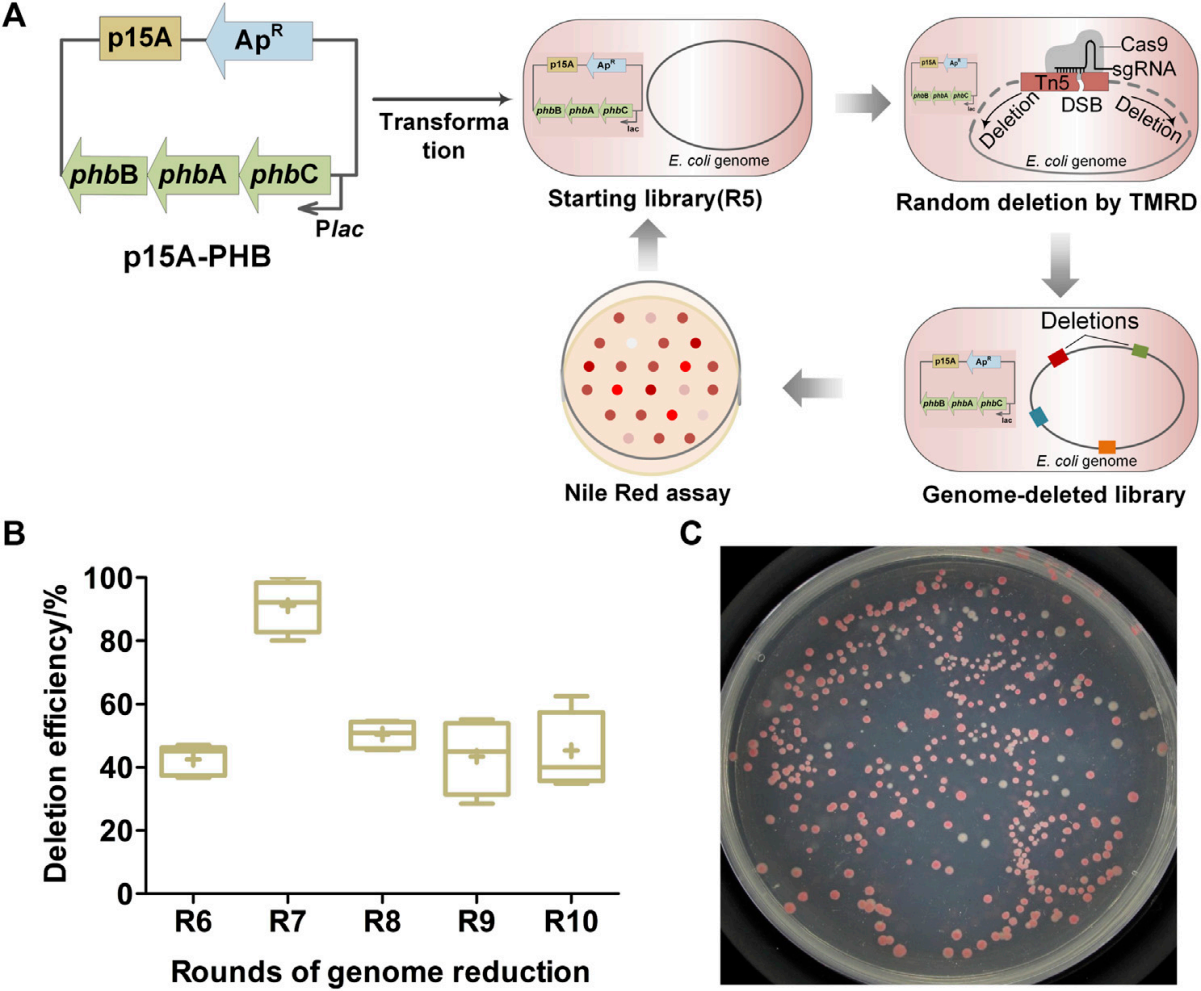
Random reduction of *E. coli* genome for PHB accumulation by transposon-mediated random deletion (TMRD) strategy. **(A)** Diagram of the coupled random genome reduction with PHB accumulation. Abbreviations: *phb*A, β-ketoacyl-CoA thiolase gene; *phb*B, acetoacetyl-CoA reductase gene; *phb*C, PHB polymerase gene; Ap^R^, ampicillin resistance gene; P_*lac*_, Lac promoter. DSB, double-strand break; sgRNA, sing-guide RNA. **(B)** Deletion efficiency of different TMRD cycles. Center lines show the medians. “+” represents the means. Box limits indicate the first and third quartiles. Whiskers show the maximum and minimum values. Six replicates were performed. At least 24 colonies were selected randomly in every replicate. **(C)** Screening of PHB competent cells by Nile red assay.

TMRD generates random genomic deletion by combining Tn*5* transposition with CRISPR/Cas9 system. Initially, a modified Tn*5* transposon carrying the sgRNA target sequence and an antibiotic gene is randomly inserted into *E. coli* genome; then, a DSB at the insertional transposon is caused by the CRISPR/Cas9 system, followed by the DSB repair *via* the endogenous alternative end-joining mechanism accompanied by DNA fragment deletions of different sizes at random positions in the genome (Ma et al., 2022). The *in vivo* PHB biosynthesis pathway is conducted by the successive action of β-ketoacyl-CoA thiolase (PhbA), acetoacetyl-CoA reductase (PhbB), and PHB polymerase (PhbC) using acetyl-CoA (AcCoA) as the precursor (Turco et al., 2020). To couple random genome reduction with PHB accumulation, we introduced the PHB biosynthetic pathway into existing TMRD gene circuits and continued to reduce the *E. coli* genome *via* TMRD method (Figure 1A). Considering that the high-copy-number ColE1 replicon has been harnessed in TMRD circuits for genome reduction, a compatible plasmid carrying the medium-copy-number replicon (P15A origin) was selected to overexpress the three key genes involving in PHB biosynthetic pathway. Initially, the p15A-PHB was electroporated in the genome-reduced library of the fifth cycle (R5) obtained in previous study (Ma et al., 2022). Then, the further genome reduction was performed using the TMRD method with slight modification (Ma et al., 2022). In each cycle, the Nile red assay was used to screen the PHB competent cells. The redder colonies that incurred genomic deletions were selected and used as the starting library for the next cycle.

Five continuous cycles of genome reduction were accomplished using R5 as starting library. It took up about 10 days for an entire cycle, the deletion efficiencies were 40%–60% on average (Figure 1B), and approximately 100 genome-deleted cells were acquired at the end of each cycle. To screen the genome-reduced mutants with high PHB accumulation, cells from the libraries acquired at the last two rounds of reduction were spreading on M9-glucose agar plates supplemented with appropriate Nile red dye (Figure 1C), and a total of 18 darker red colonies were picked out. Then, the batch cultivation was performed to determine their PHB accumulations using minimal M9 medium supplemented with glucose as sole carbon source. The 18 mutants showed different final cell dry weight, PHB contents and concentrations, with the range of 1.35–3.84 g/L, 10–25 wt% and 0.2–0.9 g/L, respectively (Supplementary Figure S1). Five of them (H2, H5, H16, H19, and H20) showed both higher final cell dry weight and PHB yields were selected for further studies (Supplementary Figure S1).

### Deletion sizes and positions analysis of the mutants *via* whole-genome sequencing

Five mutants were selected from the 18 candidates in consideration of the final cell dry weight and PHB yield and subjected to whole-genome sequencing to characterize the accumulative DNA deletion fragments. The five strains were found to have been accumulated to a total number of eight or nine DNA deletion fragments; and the total length of deletions were 230.0–270.0 Kb, encompassing 4.95%–5.82% of the genomic sequence of the parental strain MG1655 (Table 2). Among the five sequenced mutants, there were 13 deletion fragments of various lengths (1.5–82.2 Kb) that randomly distributed in different positions of the *E. coli* MG1655 genome (Table 3; Supplementary Figure S2). The number of deleted genes in these fragments ranged widely from 2 to 83, including some genes encoding insertion sequence elements, cryptic prophages, and restriction−modification system, as well as many other nonessential genes (Supplementary Table S5). The borders of most deletion events showed small micro-homologous sequences (1–11 bp) or no noticeable sequence homology (Supplementary Table S2, Table 3), implying the role of intracellular alternative end-joining mechanism (Chayot et al., 2010) in the repair of genomic double-strands breaks. In addition to genomic deletions, a few single nucleotide variants (SNVs) and small insertions and deletions (InDels) also emerged in the genomes of the five mutants (Supplementary Table S3).

**TABLE 2:** The genomic deletions of the five selective mutants.

Strains	Number of deletions	Deletion events	Total sizes/bp (ratio^a^)
H2	9	D1, D2, D3, D4, D5, D6, D8, D10, and D11	269,987 (5.82%)
H5	8	D1, D2, D3, D4, D5, D6, D7, and D8	229,758 (4.95%)
H16	9	D1, D2, D3, D4, D5, D6, D7, D8, and D9	244,962 (5.28%)
H19	9	D1, D2, D3, D4, D5, D6, D7, D8, and D12	260,608 (5.61%)
H20	9	D1, D2, D3, D4, D5, D6, D7, D8, and D13	236,324 (5.10%)

a The percentage of accumulative sizes accounts for the total length of *E. coli* MG1655 genome.

**TABLE 3:** Details of the deletion events in the five selective mutants.

No	Positions^a^	Sizes/bp	Number of genes	Length of (micro-) homology sequences^b^/bp	Descriptions
D1	4,506,953–4,589,174	82,222	83	0	Including ISs (IS911B, 60O, 30D, and 1F), restriction−modification system (*hsd*RSM, *mcr*BC, *mrr*)
D2	262,958–297,265	34,308	45	60	Cryptic prophage CP4-6
D3	2,457,372–2,503,630	46,259	48	1	Including cryptic prophage CPS-53
D4	4,302,186–4,321,787	19,602	23	0	—
D5	3,859,376–3,862,064	2,689	3	1	—
D6	2,102,833–2,123,824	20,992	20	9	Including IS5I
D7	3,581,911–3,583,427	1,517	2	0	—
D8	2,290,117–2,312,285	22,169	24	3	—
D9	1,196,220–1,211,423	15,204	26	11	Including cryptic prophage e14, restriction−modification system (*mcr*A)
D10	4,493,777–4,504,672	10,896	11	3	Including IS2K, IS4, cryptic prophage PR-Y
D11	4,069,828–4,100,677	30,850	29	5	—
D12	1,076,613–1,107,891	31,279	31	2	Including IS3D
D13	3,296,666–3,303,231	6,566	10	6	—

a Numbers correspond to the NCBI, reference genome NC_000913.3.

b The length of (micro-) homology sequences at the borders of genome double-strands breaks junction. IS, insertion sequence.

### Physiological characteristics of genome-reduced mutants

**FIGURE 2:**
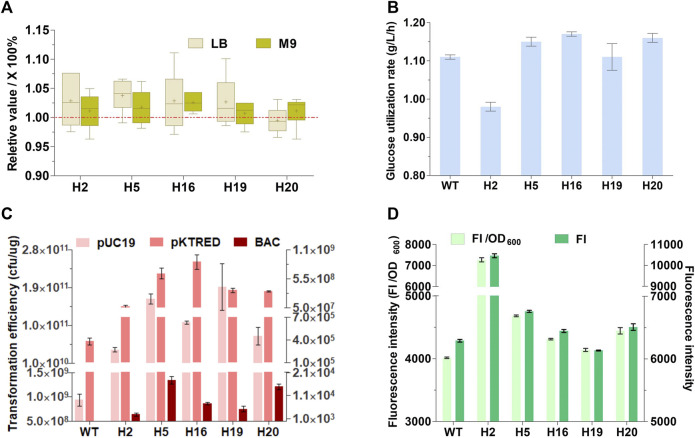
Physiological characteristics of the genome-reduced mutants. **(A)** The maximal growth rate (h^−1^) in LB rich medium and M9 minimal medium. Data presented here are the relative growth rates compared with wild-type MG1655 (WT) that are calculated as the percentage of the growth rate of mutants against the WT. The maximal growth rate of the five genome-reduced mutants and WT were measured in LB rich medium and M9 minimal medium (The data were shown in Supplementary Table S4). For each strain, six replicates were carried out. Center lines show the medians. “+” indicate the means. Box limits indicate the first and third quartiles. Whiskers show either the maximum (or minimum) value or 1.5 times the interquartile range, whichever is smaller (or larger), and the outliers are represented by dots. **(B)** Maximal glucose utilization rate in exponential phase. **(C)** Electroporation efficiency; The absolute electroporation efficiency of the WT and the genome-deleted mutants were calculated with a small multicopy plasmids pUC19, a low-copy plasmids pKTRED, and a BAC with a 92-kb fragment insertion. The electroporation efficiency refers to the total number of transformants per microgram of DNA (colony-forming units [cfu]/μg DNA). **(D)** The relative fluorescence intensity (GFP, green fluorescent protein; FI, fluorescence intensity). Values denote mean (±s.d.) of triplicates.

The growth rates and glucose utilization rates of the five selective mutants together with the wild-type MG1655 were measured (Figures 2A,B). In brief, the maximal growth rates of the five mutants approached or were higher than that of the wild-type strain both in LB rich medium and M9 minimal medium. Except for H2, all the mutants showed comparable or somewhat faster maximal glucose utilization rate in exponential phase in comparation to MG1655. These indicated that TMRD can screen suitable deletion mutants with increased cell growth and glucose utilization. An ideal chassis cell is expected to possess the excellent capacity to take up exogenous plasmids and express heterologous proteins. The electroporation efficiency of the five mutants and wild-type MG1655 was determined using a small high copy plasmid pUC19, a large plasmid pKTRED with a low-copy psc101 replicon, and a single-copy bacterial artificial chromosome with a 92-kb insertion (BAC). All mutants exhibited a significant improvement in electro-competence up to 2–4 orders of magnitude compared with the parental MG1655 (Figure 2C). The expression level of heterologous protein in the five mutants was tested using GFP as a model protein. As shown in Figure 2D, the relative fluorescence intensities of H5, H16, H19, and H20 were similar to or a little higher than that of the original strain MG1655. More significantly, H2 showed about 80% increase in the relative fluorescence intensity compared with MG1655. The results suggested that removal of some genomic regions can enhance the electroporation capacity and heterologous protein productivity.

### Increased polyhydroxybutyrate accumulation in genome-reduced mutants

**FIGURE 3:**
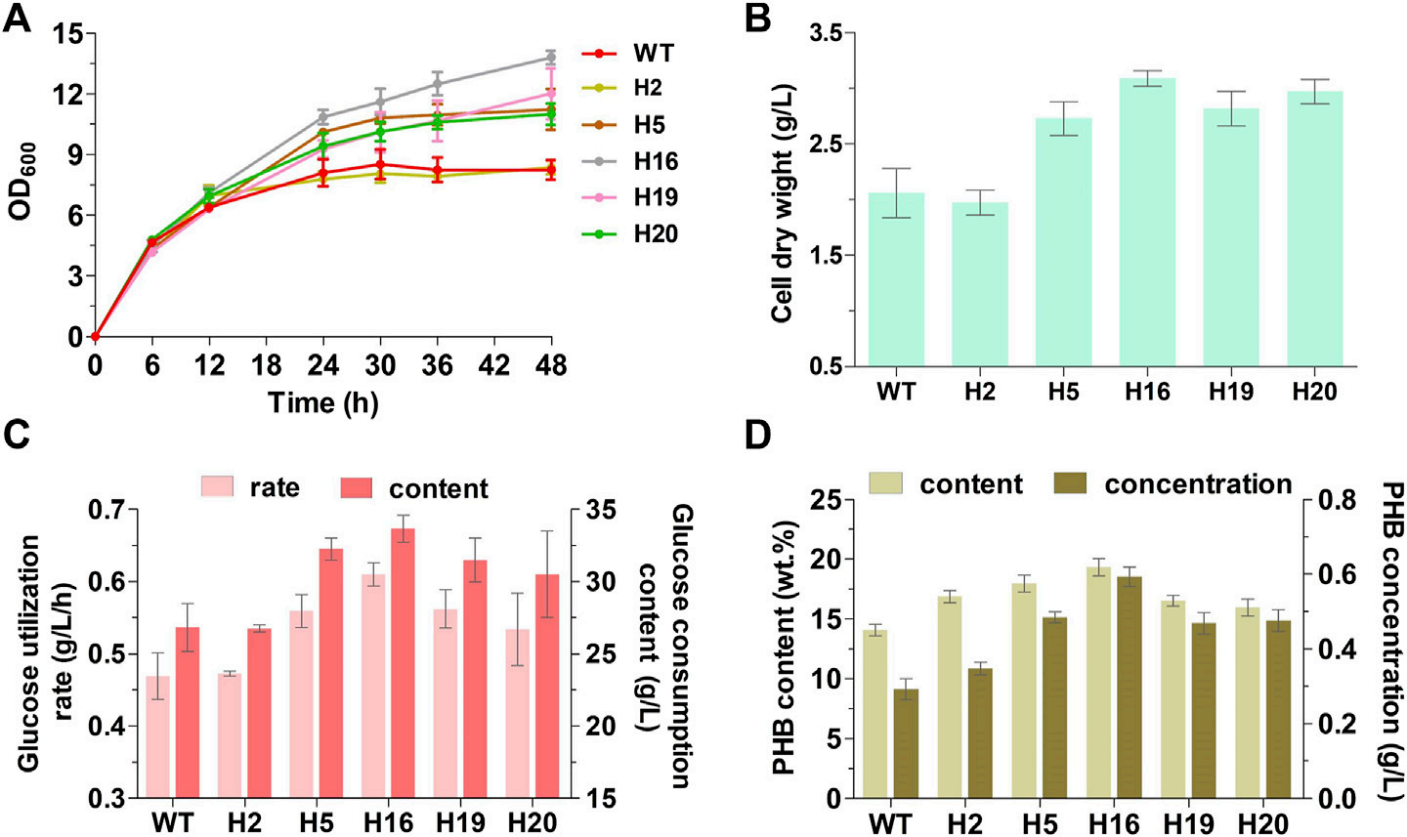
Fermentation performance of the genome-reduced mutants for PHB accumulation after 48-h cultivation. **(A)** The growth curves. Properly diluted fermentation broth was directly used for the measurement of OD_600_, which included the cell density and intracellular PHB. **(B)** The final cell dry weight; **(C)** The glucose utilization rate and accumulative consumption content; **(D)** The PHB content (g/g cell dry weight × 100%.) and concentration. WT represents wild-type MG1655. Data are expressed as means (±s.d.) from three independent experiments.

The intracellular PHB accumulation of the five mutants and the original strain MG1655 were compared by batch cultivation in M9 basal medium supplemented with glucose as sole carbon source, and the growth and glucose utilization of strains during the fermentation process were also measured. Apart from strain H2, all mutants had obvious advantages in growth and glucose utilization in comparation to wild-type MG1655, showing 32.5%–50.0% of increase in final cell dry weight, as well as 13.9%–30.1% and 13.7%–25.5% of improvement in glucose utilization rate and glucose consumption content, respectively (Figures 3A–C). The PHB content and concentration of the five mutants had enhanced up to 13.3%–37.2% and 60.2%–102.9%, respectively (Figure 3D). Despite that the cellular growth and glucose consumption of H2 were similar to that of MG1655, there still were certain degrees of increase in PHB content and concentration (19.7% and 18.9%, respectively) (Figure 3). Particularly, in H16, which displayed the fastest glucose utilization rate among all strains, the biomass (50.0% higher than that of MG1655 in final cell dry weight) and PHB yield (37.2% and 102.9% higher than that of MG1655 in PHB content and concentration, respectively) exhibited the most conspicuous improvement (Figure 3). Both mutants and MG1655 produced large amount of acetate (approx.17 g/L), and H2 (11.08 g/L) produced less acetate than other strains (Supplementary Figure S3). In the process of batch fermentation, low PHB yield was generated possibly because the key genes encoding PHB biosynthetic enzymes were expressed using a medium-copy plasmid and the weak Lac promoter. Hence, we utilized a high-copy replicon from pUC vectors and replaced the Lac promoter with the stress-induced system in which the PHB biosynthesis was induced under stress conditions (Kang et al., 2008). The PHB accumulation was evaluated in H16 together with wild-type MG1655 as control (Table 4). Results showed that the PHB productivity was improved about 3.3-fold (calculated as PHB concentration) by replacement of replicon and promoter both in wild-type MG1655 and H16. Compare with MG1655, the PHB content and concentration of H16 improved 53.2 % and 130.1%, reached 34.3 wt% and 2.6 g/L respectively, while still possessed obvious advantages in biomass and glucose utilization. Overall, we proved that random genome reduction has the potential to engineer *E. coli* to improve PHB accumulation.

**TABLE 4:** Fermentation results of H16 for PHB accumulation using high-copy-number plasmid^a^.

Strains	WT	H16	^b^ percentage of increase (%)
Cell dry weight (g/L)	5.01 ± 0.38	7.57 ± 0.18	51.1 ± 3.7
Glucose utilization rate (g/L/h)	0.54 ± 0.05	0.74 ± 0.01	36.1 ± 1.0
Glucose consumption (g/L)	29.0 ± 2.1	35.0 ± 0.71	20.7 ± 2.4
PHB content (wt%)	22.4 ± 3.5	34.3 ± 0.99	53.2 ± 4.4
PHB concentration (g/L)	1.13 ± 0.24	2.60 ± 0.10	130.1 ± 9.1
Acetate concentration (g/L)	18.41 ± 1.73	17.79 ± 0.69	/^c^

a The original strain MG1655 (WT) and mutant H16 were cultivated in M9-basal medium supplemented with 30 g/L glucose at 37°C, 220 rpm, for 48 h. The Data are expressed as means (±s.d.) from three independent experiments.

b The percentage of increase of H16 in cell dry weight, glucose utilization and PHB, accumulation compared with WT.

c Not determined.

## Discussions

In this work, we combined random genome reduction with PHB biosynthesis, and succeed in randomly and continuously reducing the *E. coli* MG1655 genome *via* TMRD method accompanied by the selection of optimal mutants for PHB accumulation. Genome reduction based on rational analysis and design has gained great progress in construction of desirable chassis for different application purposes (Sung et al., 2016; Chi et al., 2019). Owing to the inadequate understanding of genes still lacking annotation, as well as complex interactions of known genes and phenotypes, the effects of genomic deletion on cell physiology are difficult to predict and have to be tested experimentally, which is much laborious and may even cause the decay of cellular growth and performances (Ara et al., 2007; Karcagi et al., 2016; Li et al., 2016; Tsuchiya et al., 2018; Kurokawa and Ying, 2020; Liu et al., 2021). In addition, rational design method only allows the sequential removal of a single DNA fragment from a strain, and obtains one or a few genome-reduced strains in final (Posfai et al., 2006; Park et al., 2014; Liu et al., 2021). In contrast, the TMRD can randomly and continuously reduce the *E. coli* genome for a given environment, and at the same times maintained a comparable deletion rate (about 25 Kb in size per cycle every 10 days) to rational design strategy. Importantly, this method allows genomic deletions to be coupled with cellular growth, and acquire a series of genome-reduced strains with improved growth and ability to synthesize various bulk compounds.

Several random genome reduction methods based on transposition mutagenesis have been reported in some bacteria (Goryshin et al., 2003; Suzuki et al., 2008; Leprince et al., 2012; Vernyik et al., 2020; Shaw et al., 2021). Most of them have been proved the feasibility, and are inefficient to construct the chassis for industrial application (Goryshin et al., 2003; Suzuki et al., 2008; Leprince et al., 2012; Vernyik et al., 2020; Shaw et al., 2021). Our method can couple genomic deletion with PHB accumulation and demonstrated the improved accumulation of PHB by engineered *E. coli* can be achieved by random genome reduction. TMRD allows genome reduction without negatively affecting cell growth, and some genome-reduced mutants exhibited the faster glucose utilization rate, higher transformation efficiency, more biomass and PHB accumulation than that of the original strain MG1655. Importantly, the maximum increase of biomass and PHB was more than 1.5-fold and 2-fold that of original strain after batch fermentation, respectively, whatever the medium-copy plasmid or high-copy plasmid was used to express PHB pathway genes (strain H16, Table 4 and Figure 3). That suggested our method is more useful than existing random reduction methods to construct streamlined cell factory. Much effort has been done to engineer *E. coli* as an optimum chassis for PHB production with metabolic engineering strategies, and some have made great progress (Ganesh et al., 2015; Wang et al., 2019; Li et al., 2020; Boontip et al., 2021; Koh et al., 2022). The PHB productivity in this work is much lower than other researches that metabolic engineering strategies were applied to improve the PHB yields. The reason for this may be the PHB synthesis pathway is carried on a plasmid and is not directly related to genome reduction. The improved PHB accumulation possibly because the increase of cell’s glucose utilization and growth. Although low PHB yield was produced in this study, at least, we provided a new insight in generation of streamlined chassis with higher chemicals and materials productivity. In future studies, combining random reduction with metabolic engineering strategy can be used to engineer *E. coli* to further improve PHB productivity.

In general, random reduction can enrich the cells with growth fitness in different environment *via* its growth-coupled genome deletion mode (Vernyik et al., 2020; Ma et al., 2022). We observed that the five mutants harbored some same deletion fragments (D1–D8, Table 2) and similar properties (enhanced glucose utilization rate, final cell density, electroporation efficiency, and PHB accumulation). That possibly are the results of environmental screening and enrichment. The growth phase (about fifteen times in LB medium and once in M9 medium per round), Nile red assay (once per round), and electroporation steps (twice per round) incorporated in the cyclic process might act as filters to gradual screening and enrichment of the optimal deletions with fitness in the library, ultimately manifesting as the accumulation of same deletion events and the similar physiological properties of the mutants. We speculate that the increase of physiological properties (growth, glucose utilization, transformation capacity, and protein expression level) might facilitate cells to accumulate more PHB. Hence, these mutants can be easily screened *via* Nile red staining and then enriched after multiple rounds of reduction. It found that deletions of D1 (carried in all mutants) and D9 (carried in H16) contained the genes involving in restriction−modification system [*hsd*RMS (Sain, 1980), *mcr*A (Raleigh et al., 1986), *mcr*BC (Dila et al., 1990), and *mrr* (Waite-Rees et al., 1991), Table 3] that can prevent the invasion of foreign DNA elements and is a barrier to genetic manipulation in host strains (Lee et al., 2019). Since removing genes encoding the restriction−modification system greatly enhanced the transformation capacity (Wang et al., 2020), the improvement of electroporation capacity possibly because some genes that promote transformation were deleted. However, because many genes were deleted simultaneously as part of large DNA fragments, many of which are functionally unknown, and the uncertainties surrounding interactions between gene and function, it is difficult to pinpoint individual gene closely associated with the changes of physiological properties (such as, glucose utilization, growth). Moreover, the change of physiological phenotypes is more likely not caused by the deletion of an individual DNA fragment, but the combination deletion of multiple DNA segments, which make it labor-intensive to explore the causal genes. Although the relationship between genome reduction and physiological properties is hard to explain, our method has potential to obtained a series of strains with optimal genomes to produce high value-added products in the long term. In addition, the limited throughput of the final library (about 100 cells per round) decreases the diversity of deleted fragments and may cause the occurrences of repeated deletion events in different strains (Ma et al., 2022).

## Conclusion

In summary, we successfully applied the TMRD approach to construct streamlined *E. coli* hosts with improved PHB accumulation. Compared with the parental MG1655, most genome-reduced strains showed significant improvements of electroporation efficiency, biomass, glucose utilization, and PHB accumulation. Our results proved that random genome reduction can be used to construct optimal microbial cell factory and provide new insights in generating controllable chassis for different application purposes.

## Data Availability

The original contributions presented in the study are included in the article/Supplementary Material, further inquiries can be directed to the corresponding authors.
