# Supplementary material for: Random genome reduction coupled with polyhydroxybutyrate biosynthesis to facilitate its accumulation in Escherichia coli
Source: Front Bioeng Biotechnol. 2022 Aug 29;10:978211. doi: 10.3389/fbioe.2022.978211 (PMC9465206; doi:10.3389/fbioe.2022.978211)
Supplement: Supplementary file 1 [file DataSheet-1.docx]

Supplementary file

## Supplementary Figures


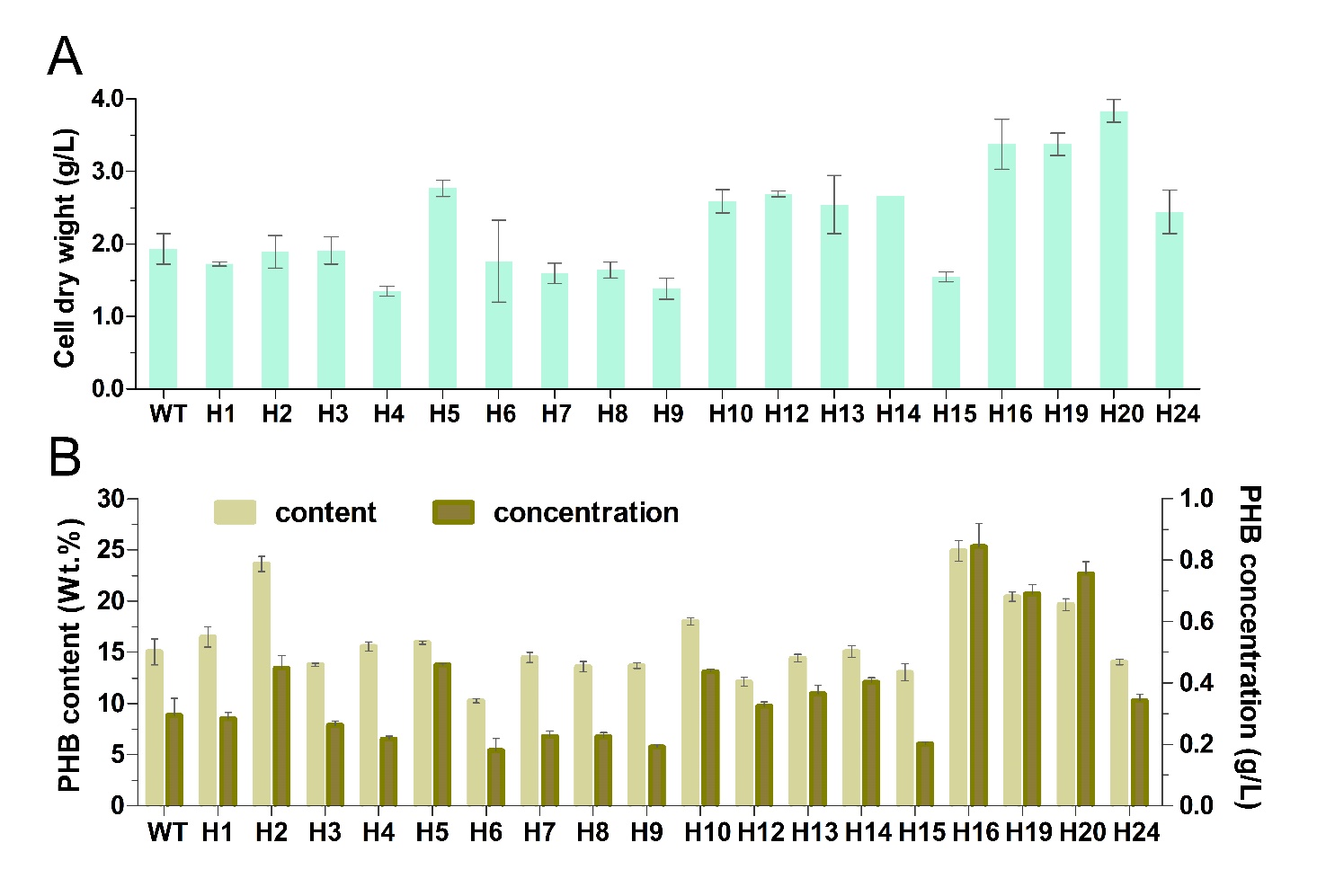


## Supplementary Figure S1. Fermentation results of the 18 candidate strains after 48-h cultivation. A. The final cell dry weight; B. The PHB content (g/g cell dry weight ×100%.) and concentration. WT: wild type MG1655. Values denote mean (± s.d.) of triplicates.

**Supplementary Figure S2.** Distribution of genomic deletions on the circular genome map of *E. coli* MG1655. The positions and lengths of D1~D13 deletion events had been indicated in blue frames in the genome. The replication origin (*ori*) and terminus (*ter*) regions are indicated in the map.





**Supplementary Figure S3.** The accumulation profiles of a**cetate** of the genome-reduced mutants after 48-h cultivation.

**Supplementary tables**

**Table S1**. Primers used in this work.

| Primers | Nucleotide sequences (5’-3’) |
| --- | --- |
| GFP-F | TTCTAGATTTCAGTTGACAGCTAGCTCAGTCCTAGGTATTGTGCTAGC |
| GFP-R | ACGAGCTCGCTTGGACTCCTGTTGATAGATCCAGTAATGAC |
| p15A-R | TACTCATATATACTTTAGATTAAGATGATCTTCTTGAGATCG |
| p15A-F | AATCAGGGGATAACGCAGGAGAGAGGGCCGCGGCAAAGC |
| Amp-R | AATCTAAAGTATATATGAGTAAACTTGG |
| PHB-F | TCCTGCGTTATCCCCTGATTCTG |
| SIP-F | AAAGGATCCGCCTGCACAAAATTCCACCGTTGCTG |
| SIP-R | TTTGGGCCCCCCCTCGAGGTCGACGGTAT |

**Table S2.** The DNA sequences at the borders of DSBs junction by the alternative end-joining mechanism.

| No | Sequences (5’ → 3’)^a^ |
| --- | --- |
| D1 | ACCATTAAAATCAAA**…---…**TACGGTGTTTCTTTG |
| D2 | GGTAGAGCAGCGCATTCGTAATGCGAAGGTCGTAGGTTCGACTCCTATTATCGGCACCATTAAAATCAAA**…---…CGCATTCGTAATGCGAAGGTCGTAGG**  **TTCGACTCCTATTATCGGCACCATTAAAATCAAA**GAGTTACCCC |
| D3 | AAATGGCGGGATCGAC**…---…C**AAGATTTTTCAAATC |
| D4 | TCACTTTCACTGATC**…---…**GTGCGCACGCACAGC |
| D5 | AATCAAACCGGTAAA**…---…**AGATACATGCAGACC |
| D6 | TGGTCTGACGGCGCT**…---…GACGGCGCT**CATCGC |
| D7 | GAACAACAAGGAAGC**…---…**GGTGATGCTGCCAAC |
| D8 | AGGAACTTCGAA**GT…---…A**GTGTTACGGTAGGT |
| D9 | GATGATGCTGCGCCA**…---…ATGATGCTGCGCCA**TATG |
| D10 | CGCCCCCATCTCTTT**…---…**TTTTTGTCTGTCTTC |
| D11 | GCTCATCGACGGCAT**…---…GGCAT**TTTCCTGCAA |
| D12 | AAATCATCCCGCA**CC…---…**CCCTACTACAAATAT |
| D13 | GGTTGGGCAGTCACC**…---…GTCACC**GCAGGGTAC |

a: Bold sequences represent the deleted sequence. Sequences underlined are micro-homologous sequences.

**Table S3**. Single nucleotide variants (SNVs) and small insertions and deletions (InDels) of the genome-deleted mutants.

| Position^a^ | H2 | H5 | H16 | H19 | H20 | Mutation | Gene | Description |
| --- | --- | --- | --- | --- | --- | --- | --- | --- |
| 2457372 | √ |  | √ | √ | √ | G→A(Q596H) | *fad*J | 3-hydroxyacyl-CoA dehydrogenase |
| 2457373 | √ |  | √ | √ | √ | A→T(Q596L) |  |  |
| 2457375 | √ |  | √ | √ | √ | A→T(K595N) |  |  |
| 3944115 | √ | √ | √ | √ | √ | A→G (intergenic, distance 2788\3013) | *yie*P\*hdf*R | Putative transcriptional regulator\DNA-binding transcriptional dual regulator. |
| 59078 | √ | √ | √ | √ | √ | △30bp (non-frameshift Deletion) | *yab*P | Putative protein |
| 2457380 | √ |  | √ | √ | √ | IS 21 bp (non-frameshift insertion) | *fad*J | 3-hydroxyacyl-CoA dehydrogenase. |
| 2457384 | √ |  | √ | √ | √ | IS 4 bp (frameshift insertion) |  |  |
| 2457387 | √ |  | √ | √ |  | IS 30 bp (stop-gain) |  |  |
| 3581910 |  | √ |  |  |  | IS 70 bp (stop-gain) | *yhh*Z | Putative endonuclease |
| 3859375 | √ |  |  |  | √ | IS 70 bp (stop-gain) | *yid*K | Putative transporter |
| 4493773 | √ |  |  |  |  | IS 63 bp (stop-gain) | *idn*D | L-idonate 5-dehydrogenase |

a: Numbers correspond to the NCBI reference genome NC_000913.3. →: base substitution; △: small fragments deletion. IS: small fragments insertion. Stop-gain: generating the stop codon.

**Table S4.** The growth rates of the genome-reduced mutants together with MG1655.^a^

| Strains | LB | M9 |
| --- | --- | --- |
| MG1655 | 2.00 ± 0.10 | 1.62 ± 0.04 |
| H2 | 2.06 ± 0.08 | 1.64 ± 0.05 |
| H5 | 2.07 ± 0.05 | 1.65 ± 0.05 |
| H16 | 2.06 ± 0.10 | 1.66 ± 0.03 |
| H19 | 2.05 ± 0.08 | 1.63 ± 0.03 |
| H20 | 1.99 ± 0.04 | 1.64 ± 0.04 |

^a^ The maximal growth rates (h^-1^) were measured using LB rich medium and M9 minimal medium. Data presented as means (± s.d.) from six replicates.
